# Ancient genomes reveal trans-Eurasian connections between the European Huns and the Xiongnu Empire

**DOI:** 10.1073/pnas.2418485122

**Published:** 2025-02-24

**Authors:** Guido Alberto Gnecchi-Ruscone, Zsófia Rácz, Salvatore Liccardo, Juhyeon Lee, Yilei Huang, Luca Traverso, Rita Radzevičiūtė, Zsuzsanna Hajnal, Anna Szécsényi-Nagy, Balázs Gyuris, Orsolya Mateovics-László, Zsolt Bernert, Tamás Szeniczey, Tamás Hajdu, Boglárka Mészáros, Marianna Bálint, Balázs Gusztáv Mende, Bryan Miller, Zainolla Samashev, Ainash Childebayeva, Leyla Djansugurova, Patrick Geary, Harald Ringbauer, Tivadar Vida, Choongwon Jeong, Walter Pohl, Johannes Krause, Zuzana Hofmanová

**Affiliations:** ^a^Department of Archaeogenetics, Max Planck Institute for Evolutionary Anthropology, Leipzig 04103, Germany; ^b^Department of Archaeology and Museology, Faculty of Arts, Masaryk University, Brno 60200, Czechia; ^c^Institute of Archaeological Sciences, Eötvös Loránd University, Budapest 1088, Hungary; ^d^Institute of Austrian Historical Research, Faculty of Historical and Cultural Studies, University of Vienna, Wien 1010, Austria; ^e^Institute for Medieval Research, Division of Historical Identity Research, Austrian Academy of Sciences, Wien 1010, Austria; ^f^School of Biological Sciences, College of Natural Sciences, Seoul National University, Seoul 08826, Republic of Korea; ^g^Institute for Data Innovation in Science, Biodata Science Center, Seoul National University, Seoul 08826, Republic of Korea; ^h^Archaeological Department, Hungarian National Museum, Budapest 1088, Hungary; ^i^Institute of Archaeogenomics, Hungarian Research Network, UN-REN Research Centre for the Humanities, Budapest 1097, Hungary; ^j^Lendület “Momentum” Bioarchaeology Research Group, Budapest 1097, Hungary; ^k^Institute of Biology, Doctoral School of Biology, Eötvös Loránd University, Budapest 1117, Hungary; ^l^Archäologischer Dienst GesmbH, St. Pölten 3100, Austria; ^m^Department of Anthropology, Hungarian Natural History Museum, Budapest 1083, Hungary; ^n^Department of Biological Anthropology, Eötvös Loránd University, Budapest 1117, Hungary; ^o^Department of Prehistory and Migration Period, Budapest History Museum, Aquincum and Archaeological Park, Budapest 1031, Hungary; ^p^Hajdúsági Museum, Hajdúböszörmény 4220, Hungary; ^q^Museum of Anthropological Archaeology, University of Michigan, Ann Arbor, MI 48109; ^r^History of Art, University of Michigan, Ann Arbor, MI 48109; ^s^State Historical and Cultural Museum-Reserve “Berel”, Zhambyl 070906, Kazakhstan; ^t^Branch of Institute of Archaeology by A.Kh. Margulan, Nur-Sultan 010011, Kazakhstan; ^u^Department of Anthropology, University of Texas at Austin, Austin, TX 78712; ^v^Center of Paleogenetics and Ethnogenomics, Institute of Genetics and Physiology, Almaty 050060, Kazakhstan; ^w^Institute for Advanced Study, Princeton, NJ 08540; ^x^Department of Historical Archaeology, Institute of Archaeology, Hungarian Research Network Research Centre for the Humanities, Budapest 1097, Hungary

**Keywords:** ancient DNA, trans-Eurasian mobility, Huns, Xiongnu, Middle Ages

## Abstract

The Huns appeared in Europe in the 370s, establishing an Empire that reshaped West Eurasian history. Yet until today their origins remain a matter of extensive debate. Traditional theories link them to the Xiongnu, the founders of the first nomadic empire of the Mongolian steppe. The Xiongnu empire dissolved, however, ~300 y before the Huns appeared in Europe, and there is little archaeological and historical evidence of Huns in the steppe during this time gap. Furthermore, despite the rich 5th to 6th centuries current era (CE) archaeological record of the Carpathian Basin, the cultural elements of connections with the steppe are limited to few findings and even fewer solitary eastern-type burials. In this study, we coanalyze archaeological evidence with 35 newly sequenced and published genomic data for a total of 370 individuals—from 5th to 6th century CE contexts in the Carpathian Basin including 10 Hun-period eastern-type burials, 2nd to 5th century sites across Central Asia and 2nd c. before current era (BCE) to 1st c. CE Xiongnu period sites across the Mongolian steppe. We find no evidence for the presence of a large eastern/steppe descent community among the Hun- and post-Hun-period Carpathian Basin population. We also observe a high genetic diversity among the eastern-type burials that recapitulates the variability observed across the Eurasian Steppe. This suggests a mixed origin of the incoming steppe conquerors. Nevertheless, long-shared genomic tracts provide compelling evidence of genetic lineages directly connecting some individuals of the highest Xiongnu-period elite with 5th to 6th century CE Carpathian Basin individuals, showing that some European Huns descended from them.

## Significance

Given their historical impact, the question of the origins of the European Huns, who they were and where they came from, has gone beyond scholarly interest and has permeated into cultural consciousness. Since the first theories that associated the Huns with the Xiongnu, academics have extensively researched and debated this topic, never reaching a consensus—except perhaps agreeing that the evidence available is very limited. In this article, we show that archaeogenomic data, if interpreted with careful archaeological and historical contextualization, can be a powerful source of information. We provide new compelling evidence on the origins of the Hun-period population, its considerable diversity and its ties to the steppe and the Xiongnu elites.

## The European Huns: Historical Background

In the 370s, the Huns suddenly burst into European history when they arrived north of the Black Sea, partially subjugating and partially displacing several ethnic groups, such as the Alans and the Goths, setting off a series of migrations into the Roman Empire. Described as a new and mysterious foe by the contemporary author Ammianus Marcellinus (1), even today the origins of the European Huns remain a matter of debate. A traditional theory, first postulated in the 18th century, links them to the Xiongnu, the founders of the first nomadic empire, which in its heyday (ca. 200 BCE to 100 CE) ruled over a variety of ethnic communities (2, 3) and stretched across Inner Asia, in the regions corresponding to modern Mongolia, Inner Mongolia, and Xinjiang. At the end of the 1st century CE, the Xiongnu Empire split into contending polities, which were later subjugated by the Xianbei and other steppe groups (4). According to most scholars, the names “Xiongnu” and “Huns” are related, although the exact nature of such linguistic connection is debated (5–7). There is little consensus as to whether there is ethnic and/or cultural continuity between the Xiongnu and the Huns (8–14). There is little evidence of Huns in the steppe between the end of the Xiongnu Empire and their appearance in Europe, and no discernible political continuity based on the written sources. Despite the substantial research on elements of the material culture that have been considered as an indication of cultural or even ethnic proximity, e.g., the so-called Hunnic cauldrons, artificial skull deformation and diadems, composite bow and arrow heads (15, 16), the archaeological evidence linking European cemeteries and settlements of the Hunnic period to Eastern Central Asia is limited (12, 17). Many researchers therefore regard the European Huns as an expanding conglomerate of steppe warriors, of mixed origins (12, 13), who had slowly moved west because of a variety of factors, including economic, climatic, and environmental ones (18). The genetic evidence from different regions of Eurasia analyzed in this article sheds light on the old question of the origins of the European Huns.

### The Archaeology of the Carpathian Basin in the Hunnic Period.

As a result of the Hun conquests in the Carpathian Basin, the Sarmatian Kingdom in the east and Roman Pannonia in the west collapsed soon after 400 CE (9, 19, 20). Many settlements and cemeteries were abandoned, and in some cases burnt destruction layers can be observed. However, other sites indicate uninterrupted use, and yet others an establishment of new, smaller settlements and cemeteries, often within former Roman or Sarmatian sites. The fate of individual communities may have differed greatly, and transformations can only be interpreted at the site level (21–23). Despite this mosaic picture, the changing society of the Middle Danube region saw the emergence of a new unified material culture under Hunnic rule. Thus, the small grave groups and cemeteries of mainly west–east oriented graves, the large number and variety of brooches and beads, polyhedral earrings, and combs in the graves are widely observed, as is the custom of artificial cranial deformation (ACD) (17, 24, 25). Instead of a strong steppe character, these cemeteries represent the 5th-century culture created by the mixed population of the Middle Danube region, closely related to that of other former Roman frontier areas.

**Fig. 1.**
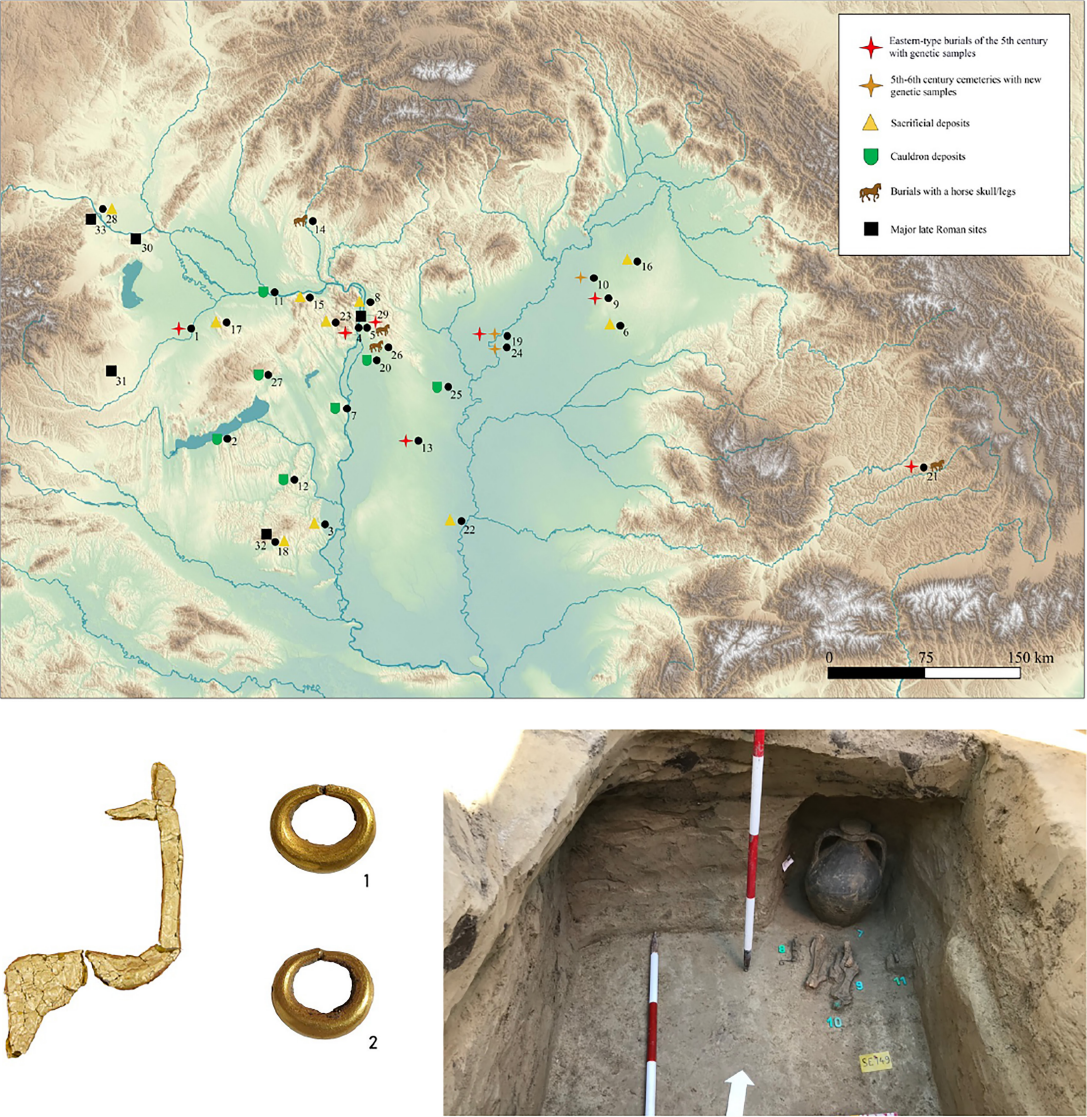
*Top*: Distribution of eastern cultural features in the Carpathian Basin in the 5th century and the archaeological sites of the newly sequenced individuals for this study. 1. Árpás (*Mursella*)-Dombiföld, 2. Balatonlelle-Rádpuszta, 3. Bátaszék-Iskola, 4. Budapest XIII, Népfürdő Street, 5. Budapest XIV, Vezér Street, 6. Debrecen-Agrár Park, 7. Dunaújváros (*Intercisa*), 8. Göd-Bócsaújtelep, 9. Hajdúböszörmény-Vidi-zug, 10. Hajdúnánás-Fürj-halom-dűlő, 11. Iža-Leányvár, 12. Kapos Valley, 13. Kecskemét-Mindszenti-dűlő, 14. Levice/Léva (1904), 15. Nyergesújfalu, 16. Nyíregyháza-Oros, 17. Pannonhalma-Szélsőhalom, 18. Pécs-Üszögpuszta, 19. Pusztataskony-Ledence, 20. Ócsa-Felsőbabád, 21. Sângeorgiu de Mureș-Kerekdomb, 22. Szeged-Nagyszéksós, 23. Telki-Annalak, 24. Tiszagyenda-Lakhatom, 25. Törtel-Czakó-halom, 26. Üllő Site 9, 27. Várpalota, 28. Vienna-Leopoldau. Major late Roman sites: 29. *Aquincum,* 30*. Carnuntum,* 31*. Savaria,* 32*. Sopianae*, 33. *Vindobona. Bottom* from the *Left*: Gold horse figurine from Árpás-Dombiföld (A181029); a pair of crescent-shaped gold earrings from Pusztataskony-Ledence (PTL013); ceramic jug and glass cup put in a niche in the grave of Budapest XIII, Népfürdő Street (NEP1).

Despite the large degree of cultural homogenization, there is some evidence obviously left by the steppe conquerors. Although very few in number, there are elements that are unprecedented in the region, and point not only to new types of artifacts but also to new habits and mentality: sacrificial cauldrons apparently used during community ceremonies and “ritual” depots related to burial rituals (26), as well as objects that indicate an equestrian nomadic lifestyle (e.g., horse tools) (Fig. 1). Several burial customs with steppe ties appeared and then vanished during the 5th century; these are primarily found in graves oriented north–south, solitary or in small grave groups and in a few cases with horse skulls and legs (Table 1). The parallels of these eastern features show different distribution patterns across the steppe. North–south orientation, offerings of ceramics or livestock set to the north, and partial animals in the graves resemble Xiongnu mortuary practices (4). North–south orientation was a common phenomenon among Hunnic-associated groups in Eastern Europe and Central Asia (27, 28), as well as in other Eastern European and Pontic cultural contexts, including late Sarmatian burial sites and cemeteries of the Chernyakhiv–Sântana de Mureş culture. Thus, its appearance in the Carpathian Basin in the first half of the 5th century cannot be linked exclusively to Hunnic groups, but generally to a new eastern influence (29). The eastern connections of the material culture are mostly limited to the Pontic steppe and the Caucasus region (e.g., crescent-shaped earrings) (30, 31), and no uniquely East Asian artifacts can be detected (32). Eastern features are not uniformly present in the burials and often co-occur with local elements (e.g., deposition of ceramic jugs and glass vessels in the grave pit according to Roman customs). These burials (hereafter “Hun period eastern-type-burials”) are quite rare and scattered throughout the Carpathian Basin (9, 33) (Fig. 1).

**Table 1.** Archaeological characteristics of the 5th-century eastern-type burials from the Carpathian Basin discussed in this study

Site	Gen ID	Sex	ACD	Burial form	Orientation	Animal bones	Weapon	Jewelry/dress	Ceramics/glass (head)	Other
Árpás (Mursella)- Dombiföld	A181029	M	–	Solitary, in a Roman building	NNW-SSE	Sheep sacrum, bovine lower leg	–	Gold belt/shoe buckles	Jug, glass cup, ceramic cup	Gold animal figure, kettle, iron knife, tweezers
Budapest XIII, Népfürdő Street SR172	NEP2	M	–	In a small group of graves, next to a Roman fortress; a 2nd skeleton above it	N-S	Pork thigh	Long sword, gilded, pearl	Silver belt/sword belt/shoe buckles	Jug, glass cup in a small niche	Nagaika (lash), bone comb
Budapest XIII, Népfürdő Street SR146	NEP1	M	–	Placed on top of NEP2 in the same grave pit	NW-SE	–	–	–	–	–
Budapest XIV, Vezér Street	VZ12673	M	–	Solitary	NE-SW	Horse skull with 3 vertebrae	–	Iron belt/shoe buckles	Ceramic fragment	Bronze bell, iron bell, fragments of a gold diadem, iron bit, knife
Hajdúböszörmény-Vidi-zug Grave 55/58	HDB001	M		2 Hun-period graves dug into a prehistoric kurgan	N-S	Pits with animal remains around the grave	–	Gold belt/shoe buckles	Jug	Iron knife, iron tool
Kecskemét-Mindszenti-dűlő SNR 2785	KMT-2785	M	ACD	Solitary, sidewall niche	N-S	–	Short sword	Gold belt/shoe buckles, crescent-shaped earrings	jug	Roman bronze coin, iron knife with gold plate
Pusztataskony-Ledence Grave 270/337	PTL013	F	ACD	Solitary	N-S	–	–	Crescent-shaped earrings	Jug, pot	Amber bead
Sângeorgiu de Mureș Grave 1	MSG-1	M	ACD	Grave group of 2 graves	N-S	Horse skull and lower legs	Gold mount of a short sword?	Iron buckle	Jug, glass goblet	Antler comb, bone tool

After the fall of the Hun Empire, the eastern half of the Carpathian Basin became part of the Gepidic kingdom (454 CE). This did not, however, represent a sudden break in the material culture. Some of the sites, especially the smaller cemetery groups and loosely structured burial grounds, were used in the transition period from the Hunnic to the Gepidic periods. Subsequently, during the latter part of the 5th century, larger “row grave cemeteries” emerged, following the prevalent patterns in early medieval Europe. Other traditions from the Hunnic period, for example, the habit of ACD, did not completely disappear (29). Therefore, it appears from the archeological evidence that the mixed Hun-period population persisted (at least in part) during the time of the Gepidic kingdom.

The goal of this study is to investigate the potential connections between the Carpathian Basin during and after the Hun period and the populations from the preceding centuries in the Central and Eastern Eurasian Steppe via the analysis of ancient genomic data. Specifically, we want to assess whether it is possible to identify evidence of direct genetic descendants between the Xiongnu period population in the Eastern Eurasian steppe and the Carpathian Basin population during and after the Hun period. To this end, we present new genome-wide data for 35 individuals (Dataset S1): four from 5th-century Hun period eastern-type contexts, one from a solitary grave (Pusztataskony, PTL013), one from a small group of graves (Hajdúböszörmény, HDB001) and two from a possible double burial (Budapest XIII, Népfürdő street, NEP1 and NEP2); 19 individuals from cemeteries dated to the 5th to 6th centuries (Tiszagyenda or TGB/TGH, Pusztataskony or PTL, Hajdúnánás or HNF), that showed mixed Central or East Asian ancestries within a larger sample set (N = 228) and 12 individuals from the Xianbei/Hun period site of Berel located in the western slopes of the Altai mountains (Berel_Altai_Xianbei_Hun_P 2nd to 5th c. CE) (Datasets S5 and S6). We coanalyze the new data with published genomic data from the late 4th to late 6th centuries in the Carpathian Basin, data from the Xiongnu period (3th c. BCE to 1st c. CE) in the Eastern Eurasian steppe, and finally data from the Central Asian steppe following the Xiongnu period (2nd to 6th centuries).

## Results

### Description of the Sites and Individuals Selected.

We compiled an ancient genomic dataset consisting of a total of 370 ancient individuals (Dataset S2), of which 275 met the criteria for identical by descent (IBD) segment sharing analyses (*Materials and Methods*). For the sake of description, we can group these individuals into three main geotemporal categories based on chronological order: 1) individuals from Xiongnu contexts (N = 80) from the Eastern Eurasian Steppe that can be further divided into early Xiongnu period (209 BCE to 50 BCE) and late Xiongnu period (49 BCE to 98 CE); 2) individuals from Central Asian contexts dated to the 2nd to 6th centuries (N = 63); and 3) individuals from late 4th to 6th century contexts in the Carpathian Basin (N = 143). For genetic comparison, we also included other early medieval East Asian contexts (~1st to 8th c. CE) in the analyses, among them, sites in Xinjiang south of the Mongolian steppe and east of the Tian Shan Mountains, as well as sites in Guangxi, in continental China and in Taiwan.

In more detail, the Xiongnu-group individuals were retrieved from 34 Xiongnu-period sites scattered across the Eastern Eurasian Steppe, some associated with late Xiongnu-period imperial elites (i.e., TAK001, TAK002, and DA39; lateXiongnu_P_elite 1st c. BCE 2nd c. CE), others with local elite or general population contexts (earlyXiongnu_P 2nd−1st c. BCE and late Xiongnu_P 1st c. BCE 2nd c. CE) (2, 3, 34).

The Central Asia group individuals include data from broad areas and different archaeological contexts. We included sites in southern Central Asia from 2nd to 6th century nomadic contexts in the Tian Shan mountains (TianShan_Hun_P 2nd to 6th c. CE) and the neighboring sedentary contexts associated with the Kangju Kingdom (Kangju 2nd to 4th c. CE) (34, 35). We also included data from sites in the northern steppes mostly coming from solitary burials from 3rd to 5th c. CE nomadic contexts, scattered along a wide geographic area: the 4th to 5th century elite burial of Aktobe/Kurayly (KRY001) (35, 36), a north–south oriented grave with a horse skull, a saddle, and numerous gold objects (37). The 5th-century solitary burial of Halvay (DA27), dug into a Bronze Age kurgan is also oriented to the north, furnished with a horse skull, a sheep skull, arrows, and a bow (38) and other two 3rd-5th c. solitary burials (Early_Medieval_Nomad 3rd to 5th c. CE). These burials are contemporaneous with and culturally closely related to Hun-period graves discovered even further west in the Volga and the Pontic steppe region as well as in the Carpathian Basin (9).

Additionally, the Central Asia group includes 18 Berel_Altai_Xianbei_Hun_P 2nd to 5th c. CE (or BRE) individuals (12 new and 6 published). These 2nd to 5th century nomadic burials were discovered within an Iron Age 3rd c. BCE elite Pazyryk archaeological context (35, 39). The east-west orientation of the graves, the intensive use of stones in the grave structures and the burials of whole horses differ greatly from the north–south oriented solitary graves buried with horse skulls/skins in the Eurasian steppe. The burial customs in Berel seem in line with the previous Pazyryk traditions, even if separated from them by the centuries-long Xiongnu era hiatus. However, the rectangular body-sized stone cist or carved out log coffin and the body stretched supine both resemble the Xiongnu traditions. Thus, the Hun-Xianbei period burials surrounding the large Pazyryk mounds at Berel are an intriguing cultural mix of practices that emerged across the Inner Asian grasslands during the Xiongnu period along with some practices particular to the Altai that seemingly persisted or re-emerged in the Berel region after the collapse of the Xiongnu regime.

The Carpathian Basin group includes individuals from various archaeological contexts dated from the late Sarmatian/Hunnic to the Hunnic and Gepidic periods (4th to 6th c. CE). These contexts are 1) cemeteries of the latest phase of the Sarmatian period in the Carpathian Basin (4th to early 5th c. CE), 2) scattered graves belonging to larger burial compounds from the Hun and Gepidic periods (5th to 6th c. CE), 3) Gepidic-period row grave cemeteries (2nd half of 5th to 6th c. CE) (36, 40–43). Among these are the 20 individuals with East Asian admixture newly sequenced for this study. 4) The remaining 10 individuals belong to typical Hun-period eastern-type burials of the 5th century, the four newly sequenced individuals described above (PTL013, HDB001, NEP1, and NEP2) and six recently published (Budapest XIV or VZ12673, Kecskemét or KMT-2785, Árpás or A181029, Sângeorgiu de Mureș or MSG-1, and 2 burials of Czulice Site 21, czu001 and czu002) (35, 36, 43, 44). Czulice site is located in present-day southern Poland, north of the Carpathian Basin, but for simplicity, we will consider them in the Carpathian-Basin group. These show several emerging eastern features, including the solitary position and north–south orientation of the grave pits, the burial of a horse skull/horse skin, vessels—mainly ceramic jugs—placed next to the head, and some eastern object types, such as crescent-shaped gold earrings, and in three instances artificial cranial deformation (28, 45–47). It is also common to find these burials within settlement remains or burial grounds of earlier sites, for example within a Sarmatian settlement, a Roman building or even a prehistoric kurgan (28).

### Genomic Composition and Close Relatedness within the Geotemporal Groups.

**Fig. 2.**
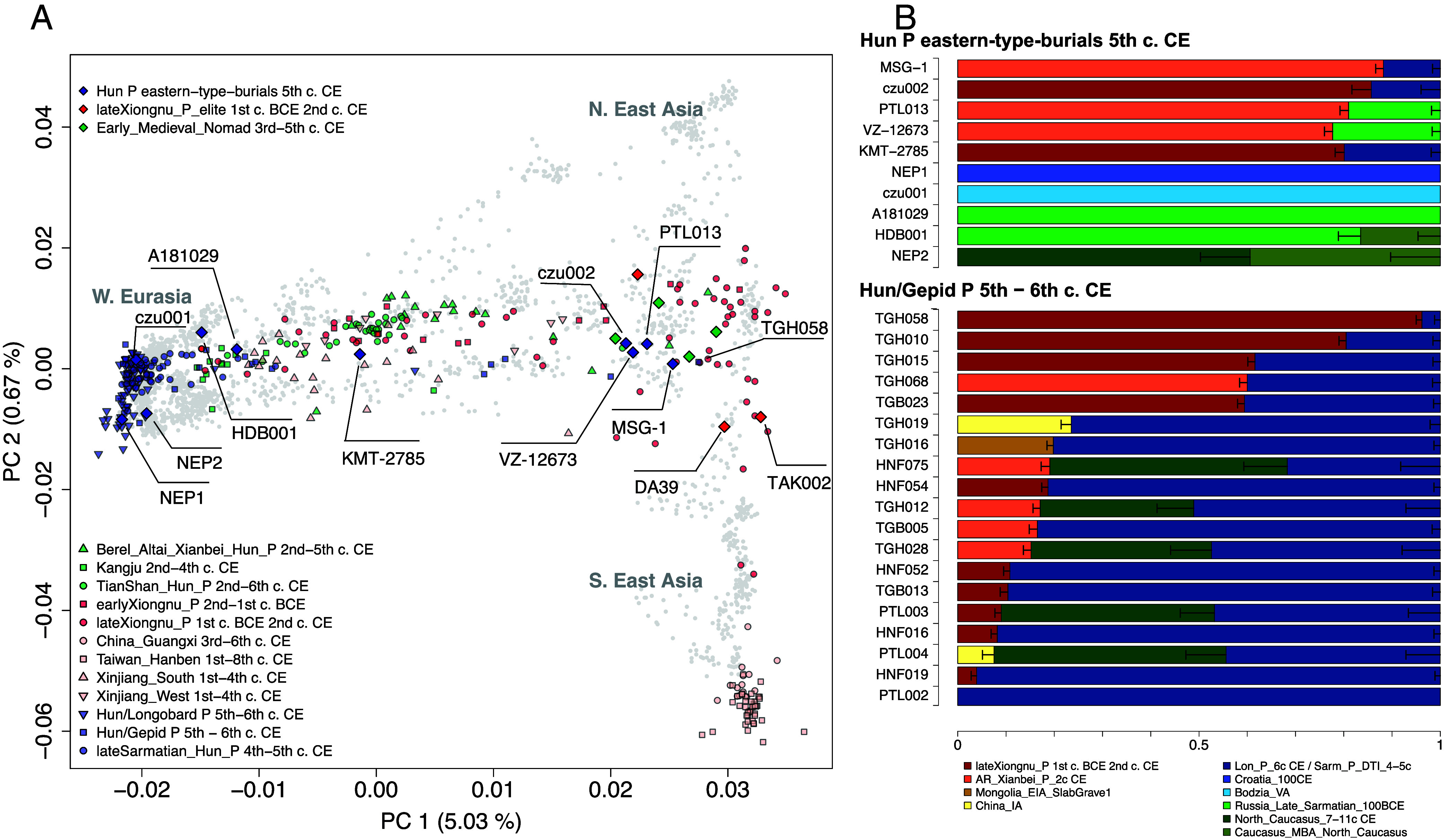
(*A*) Eurasian PCA: the 370 ancient individuals analyzed in this study are projected on top of a PCA calculated with present-day Eurasian populations. Most relevant individuals’ IDs are pointed out. (*B*) Best qpAdm models (*P*-value > 0.01) of the Carpathian Basin individuals from the 10 Hun period eastern-type-burials (*Top*) and from the Hun or Gepid period (5th to 6th c. CE) context presenting East Eurasian/Steppe admixture signals.

In terms of genomic ancestry composition, the Xiongnu-period individuals as well as the 2nd to 6th century Central-Asian individuals (green) are distributed along a wide heterogeneous East to West Eurasian admixture cline (Fig. 2) as described in previous articles (2, 3, 48). Most of the Carpathian-Basin individuals (shown in blue) from the late 4th to the 6th century do not show any signs of East/Central Asian genetic admixture, carrying only European ancestries (Fig. 2*A*). Between these contexts 19 individuals show varying amounts of East Asian admixture. Among these, the individuals TGH058, TGH010, TGH015, TGH068, and TGB023 from poorly furnished 5th to 6th century graves within the site of Tiszagyenda have the highest amounts of Eastern Eurasian ancestry. Lower amounts of eastern ancestry are also found in individuals of the Gepidic-period Hajdúnánás cemetery (Fig. 2*B* and Dataset S3). Among the 10 individuals found in Hun period eastern-type-burials, eight show varying amounts and dates of West/East Eurasian admixture. There is a high heterogeneity in west to east admixture dates and admixture proportions between all of these admixed individuals. Most of them carry the same North East Asian ancestry (i.e., ANA) found in many Xiongnu- or Xianbei-period individuals (2, 3, 48) (Fig. 2*B*). The Hun-period eastern-type-burial individuals tend to have older admixture dates closer to the Xiongnu period (i.e., PTL013, VZ-12673, KMT-2785, MSG-1, and czu02), while the other 19 eastern outliers tend to have more recent admixture dates closer to the Hun period (*SI Appendix*, Fig. S1 and Dataset S4). This shows that the incoming steppe-related individuals already carried mixed ancestries and suggests additional postarrival admixture within the Carpathian Basin with populations carrying European ancestries. Furthermore, the individuals from the Hun period eastern-type-burials of Árpás/A181029, Hajdúböszörmény/HDB001, and Budapest Népfürdő street (NEP2) have very different genomic profiles with respect to the other individuals (Fig. 2*B*). The first can be modeled as deriving all its ancestry from a Sarmatian/Scythian Central Asian steppe gene-pool which is itself a result of a more ancient admixture layer along which Iron Age Scythian-related populations were laying on (Russia_Late_Sarmatian_100BCE) (49). HDB001 has a very similar genomic ancestry but requires additional ~16% from other ancient Caucasus sources. This is consistent with a much older admixture date (~50 to 100 generations, ca. early first millennium BCE) estimated for A181029 and HDB001 with respect to the other individuals described before (*SI Appendix*, Fig. S1 and Dataset S4). NEP2 instead can be modeled as deriving its ancestry from a mix of ancestry sources from the Caucasus (Fig. 2*B*). Only two Hun period eastern-type-burial individuals (czu001 and NEP1) present a northern and a southern European ancestry respectively (Fig. 2*A*). NEP1 provided a working model with one of the southern European sources tested (Croatia_100CE), a Roman period group from present-day Croatia (50). By contrast, czu001, being at the northernmost position on the cline of variability for the 5th to 6th- century Carpathian Basin, provides a working model with the more northern ancestry source of Bodzia_VA [a Viking Age site in present-day Poland (51)] (Fig. 2*B*). It is noteworthy that both of these individuals were part of a double burial: NEP1 was without grave goods but buried on top of the richly furnished NEP2; czu001 also lacked grave goods, and was found buried on his stomach next to the richly furnished czu02 (44). These results demonstrate the high genetic diversity of the population living in the Carpathian Basin during the Hun period, especially among the Hun period eastern-type-burial individuals, and a mixed ancestry background among the people arriving from the steppe.

**Fig. 3.**
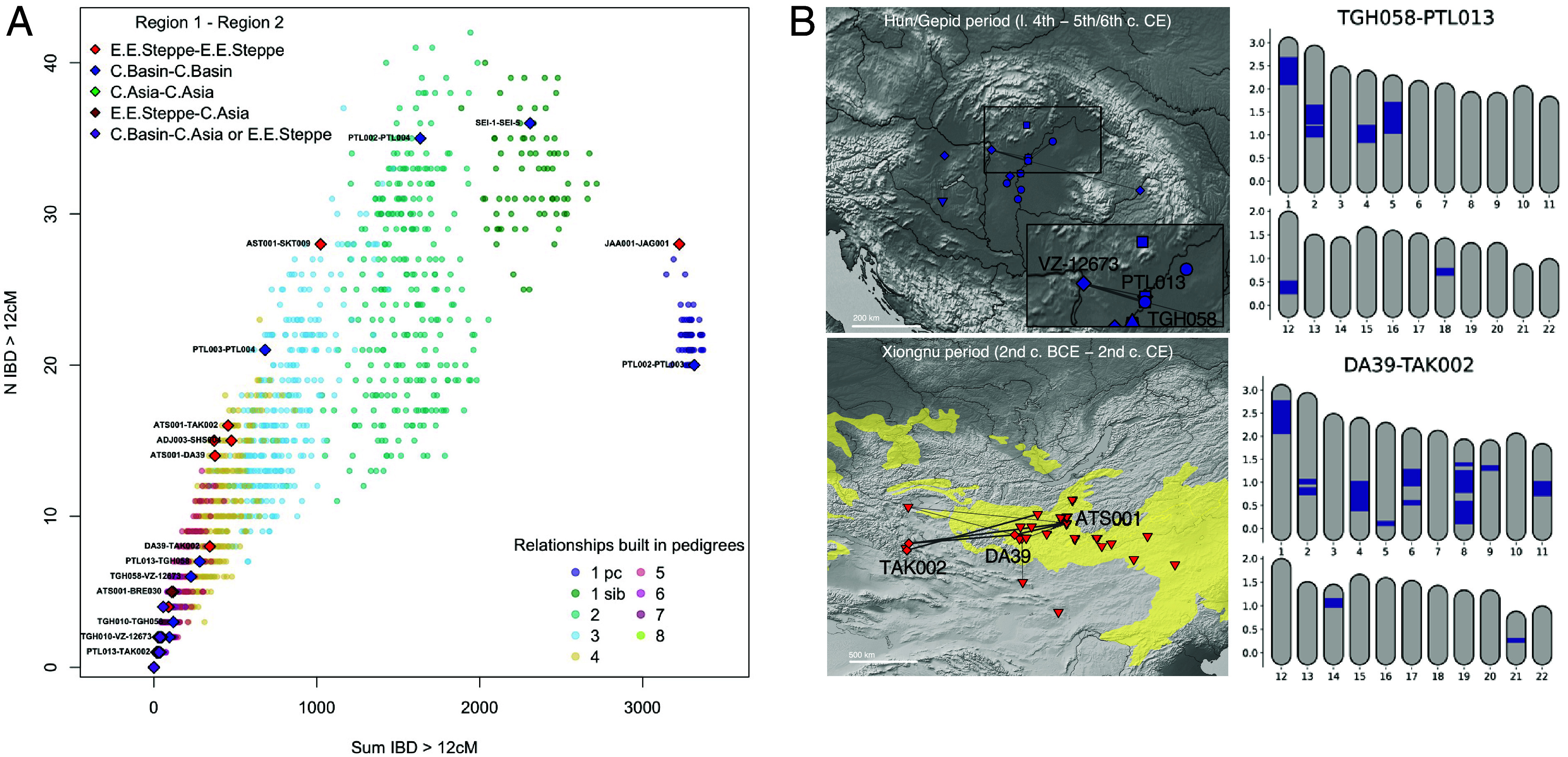
(*A*) Background colored dots represent the distribution of IBD-sharing between a reference set of ~300 closely related individuals color coded according to their degree of relation inferred from the pedigrees (42), from 1st degrees parent–child (pc) and siblings (sib) to the 8th degree of relation. On top of this distribution the plotted colored diamonds are the top IBD-sharing pairs among the 275 trans-Eurasian individuals analyzed in this study. Different colors of the diamonds represent the different combinations of macroregions of the pairs of individuals plotted: Carpathian Basin (C.Basin), Eastern Eurasian Steppe (E.E.Steppe), Central Asia (C.Asia). (*B*) Maps showing the intraregional Carpathian Basin (*Top*) and Mongolian Steppe (*Bottom*) IBD sharing and the karyotype plots (i.e., the 22 autosomal chromosomes) of two pairs as example, with the IBD tracts shown in blue.

We then estimated the pairwise sharing of IBD DNA segments and found numerous pairs of individuals connected by IBD segments, suggesting close relatedness, both within and between each of the three geotemporal groups (Fig. 3). The highest IBD sharing pairs are found within each of the groups and include some first- and second-degree relatives that were previously identified (Fig. 3*A*) (2, 43). We also identified relatively closely related individuals such as a trio involving two Hun-period eastern-type-burials of PTL013 and VZ-12673 (5th- to 7th-degree relatives) and TGH058, a female individual from a poor and disturbed grave within the 5th to 6th century settlement of Tiszagyenda (4th- to 5th-degree related to PTL013 and VZ-12673) (Fig. 3*B*). The close biological relatedness between the two individuals in the Hun-period eastern-type solitary burials and TGH058 evidences that either these ancestral connections were associated with diverse cultural markers, or that a Hun-period burial was located within a Gepidic cultural context. Another significant finding involves three Xiongnu-period individuals related to each other in the 3rd to 5th degree, belonging to two imperial elite burials from the sites Gol Mod 2 (DA39) and Takhiltyn Khotgor (TAK002) and a local elite burial from the site of Atsyn Am (ATS001) located in a range between 350 and 1,000 km from one another (Fig. 3*B*). In fact, in the Xiongnu period a total of six pairs of closely related individuals (1st to 4th degree) are found across different sites (Fig. 3*B*). Finding close relatives across these large distances is probably indicative of the high mobility and nomadic nature of the Xiongnu empire and/or of unions among the Xiongnu elite.

### Trans-Eurasian Genomic Connections.

**Fig. 4.**
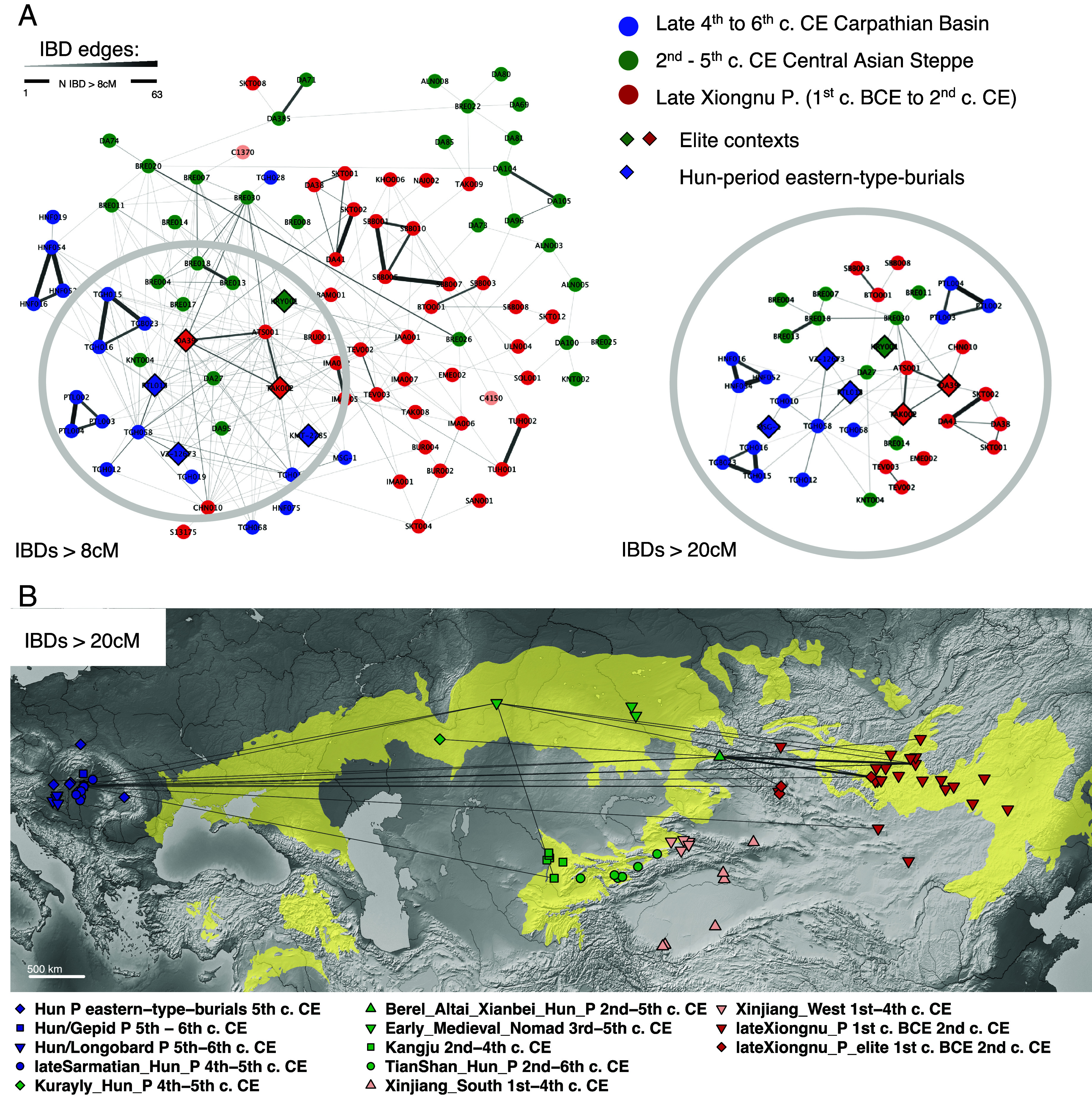
(*A*) Networks of trans-Eurasian IBD sharing between the Individuals analyzed in the study, considering IBD tracts >8 cM (*Left*) or >20 cM (*Right*). (*B*) Map showing the geographic distribution of the trans-Eurasian IBD sharing depicted in the *Top Right* network (>20 cM).

Our most striking finding is the haplotype IBD-sharing that connects the three geotemporal groups, even when considering only pairs with ≥3 shared IBD tracts between 8 cM and 12 cM and all >12 cM in length to minimize false positive discovery (52) (Fig. 4*A*). These form a network of 97 interconnected individuals from the late Xiongnu period in the Mongolian steppe, through the 3rd- and 5th-century Central Asian steppe burials until the late-4th to 6th-century Carpathian Basin (Fig. 4). The core of the network, where most of the trans-Eurasian connections are found, contains around 20 individuals including the closely related Carpathian Basin and Xiongnu-imperial-elite trios described in the previous section, another eastern-type burial (MSG-1) and the two Central Asian Steppe solitary burials dated to the Hun period, Aktobe/Kurayly, and Halvay (KRY001 and DA27) (Fig. 4*A*). Interestingly, the connections between the Central Asia group and the other two groups are mostly mediated through a limited number of sites and burials across the Central Asian steppe: three solitary burials of KRY001, DA27, DA95, and 10 Berel_Altai_Xianbei_Hun_P 2nd to 5th c. CE. While these solitary graves are culturally related to the Hun-period eastern-type burials in Europe, the same cannot be claimed for the Berel site. The different burial traditions make the interpretation of these IBD links lean toward a common source rather than a direct connection between the population of Berel and the Hun-period Carpathian Basin, as from the genetic results both scenarios are possible. We do not find IBD connections with the southern part of the Central Asian Steppe; the only exception is represented by an East Asian ancestry outlier individual (KNT004) found in the Otrar Oasis sedentary context within the Kangju Kingdom area (2nd to 4th c. CE) (35).

Furthermore, no long (≥20 cM) and very limited shorter (≥8 cM) IBD connections are detected between the Xiongnu period or the late 4th to 6th c. Carpathian Basin and individuals from nomadic contexts of the Tian Shan mountains (2nd to 6th c. CE; Fig. 4 and *SI Appendix*, Fig. S2). This confirms the archaeological observation that, although groups from Tian Shan are usually described in the literature as “Huns” (34), their material culture shows no affinity with the European Hun-period archaeological finds. Nevertheless, our understanding of population shifts in Southern Central Asia during the 4th and 5th centuries is limited because we lack individuals of either steppe-type solitary burials or sites considered to be “Hunnic” (e.g., belonging to the Kidarite or Hephthalite realms). We also found very limited IBD sharing between the other early medieval East Asian sites and individuals from the neighboring Xinjiang region (Fig. 4*B* and *SI Appendix*, Fig. S2), suggesting that the amounts of IBD sharing observed between the Carpathian Basin, Central Asia, and Xiongnu period individuals cannot be ascribed to general sharing of East Asian genomic components. Furthermore, when we modeled the pattern of IBD sharing between the interconnected individuals from the late Xiongnu period and the late 4th to 6th c. Carpathian Basin individuals, we found a split time of ~500 y, corresponding to the median date difference that separates the Hun and Xiongnu periods (*SI Appendix*, Fig. S3 and section S1). This shows that some late Xiongnu individuals (including two found in imperial elite burials) are either the direct ancestors of some Hun-period individuals or are genealogically connected to their direct ancestors by a few generations. It is tempting to speculate that there were a handful of elite Xiongnu lineages or “families” driving the longer distance moves over generations. We cannot be sure, however, that this biological ancestral tie had any cultural or social meaning attached to it, especially after so many years. Other plausible interpretations why elite individuals have more IBD connections could be related to social practices of steppe clan marriages (42) or other social conditions (e.g., higher resources/quality of life) that determined a higher chance of having a higher number of biological offspring that spread the genetic signal over generations.

## Discussion

In line with results from previous studies, we confirm that some of the individuals found in Hun-period eastern-type burials in Europe carry varying amounts of northeast-Asian ancestry (35, 36, 43, 44). Similar admixed genomic ancestry profiles were commonly found across the whole Eurasian steppe in the preceding millennia (2, 3, 34, 35, 53, 54). Therefore it is not obvious to connect them to specific preceding steppe archaeological cultures and people. Here, by adding new data and refined haplotype IBD analyses, we find compelling evidence of direct genetic descent lines connecting some of the highest elite late Xiongnu period individuals with some of the few archaeologically defined Hun-period eastern-type-burials in the Carpathian Basin, and some other individuals from other 5th to 6th century contexts in that region carrying genetic signatures of East Asian admixture.

Furthermore, by surveying data for a total of 371 individuals from other 5th to 6th century contexts from the Carpathian Basin (143 included here) we find only 26 individuals (6%) with signatures of North East Asian or Steppe admixture. This includes 8 out of 10 individuals from Hun period eastern-type-burials. These graves are few in numbers, all solitary or in small grave groups, and geographically scattered mainly east of the Danube (Fig. 1). Therefore, apart from these direct descent lines linking these individuals with eastern ancestry, both archaeologically and genetically we do not find evidence for the presence of larger eastern/steppe descent communities in this time period.

Overall, the results of this study allow several conclusions about the history of the European Huns. First, the population of the Hun realm in Europe was genetically highly heterogeneous. This is especially true for the individuals found in the Hun-period eastern-type burials. These could be interpreted as belonging to the military elites and their families, whom contemporaries regarded as Huns, and whose ancestries spread along the entire East-West Eurasian genetic cline. Second, however, there are clear indications that some of the individuals buried in 5th-century East Central Europe had IBD connections to earlier burials in the Central and Eastern Eurasian steppes: including, but not exclusively, the former lands of the Xiongnu, which proves that some European Huns were descended from there. These connections also involve individuals buried west of the former Xiongnu lands (i.e., the Berel site west of the Altai mountains), where parts of the Xiongnu may have withdrawn after their defeat (5). Burials west of the Altai mountains have IBD connections to Europe, but little in common archaeologically. Sites still further west in Northern Kazakhstan (such as Aktobe/Kurayly), display some archaeological parallels with Hun-period Central Europe. These solitary burials may be a trace of the same Hun movement that swept through Europe in the 4th to 5th centuries. Third, we could not establish any substantial direct connections between either the Xiongnu or the European Huns and the region of the Tian Shan and the oasis of Otrar in Southern Kazakhstan. However, this remains a preliminary conclusion that should be tested with data from a wider region of Southern Central Asia.

We can conclude that the migration of the Huns in the 370s into Eastern Europe differed from that of the Avars two centuries later (36, 55). The Avar core group in the 6th century fled to Europe directly after the defeat of the Rouran Empire in East Asia, and had predominantly Eastern and Central Asian ancestries. The European Huns of the 4th to 5th century are temporally more distant to the latest Xiongnu populations of the 1st/2nd centuries, and were genetically and culturally diverse. The far-reaching IBD connections, but also the diverse ancestries, the wide range of admixture dates and archaeological differences indicate a more complex process of mobility and admixture than a one-off long-distance migration.

## Materials and Methods

### History of the Huns in Europe.

The abrupt appearance of the Huns in the Pontic-Caspian Steppe in the 370s marks the beginning of the so-called Migration Period. Their clash with the Alans and the Goths triggered a series of events that changed the history of both the *barbaricum* and the Roman Empire (56–58). Once they had subdued the Alans, the Huns proceeded to attack the Goths, who could not resist them and sought asylum in Roman territory. In the beginning, the Huns lived in a loose political structure, but over the course of a few generations, they established a hierarchical division of power, extended their central control of a wide territory north of the Roman border, integrated a consistent number of non-Hunnic individuals into military and administrative cadres, and thus became a multiethnic and multilingual empire.

Even though the shifting territorial boundaries of their empire can hardly be ascertained, the Huns ruled over numerous ethnic groups in Eastern Central Europe and provided an alternative model of rulership to the Roman Empire (10, 59, 60). After c. 400, the center of the Hunnic polity is thought to have moved from the Pontic region to the Lower Danube and subsequently to the Carpathian Basin. In this period, the Huns played an increasingly important role in Roman politics, to the point of instrumentalizing internal strife in the empire for their own benefit (61), and became a dangerous if not existential threat to the Roman Empire under the reign of Attila. In the 440s and 450s, Attila ruled over a thriving multiethnic empire, as evidenced by the first-hand account of Priscus (62), and launched several devastating raids into the Balkan Peninsula, Gaul, and Italy (63). However, once Attila died, his empire disintegrated within a few months: A struggle for succession led to war between Attila’s sons, and two coalitions including the former allies and picked men (*logades*) of Attila clashed at the Battle of Nedao in 454. As a result, Gepids and Goths conquered core regions of the Carpathian Basin, and the Hunnic Empire dissolved into a mosaic of competing barbarian polities. Attila’s sons were pushed out toward the Pontic steppes. Although Hun dominion was only an episode in the gradual collapse of the Western Roman Empire, Attila acquired legendary status in the sources and remains one of the best-known historical figures of the period until today.

### Archaeological Description of the Sites with Newly Sequenced Individuals from the Carpathian Basin.

#### Budapest, District 13, Népfürdő Street (H).

The two Hun-period burials were found at 47 Népfürdő Street in the 13th district of Budapest. The site is located opposite the Aquincum legionary fortress built along the right bank of the Danube, and outside the southern wall of the Roman fort near the Rákos Stream. The Budapest History Museum has been carrying out archaeological work in the area since 2018, which has revealed Roman and Hun period remains, as well as Late Copper Age, Early Bronze Age, Avar, Árpádian Age, and Late Medieval features. The archaeological and anthropological examination of the Hun-period burials (SR146, SR172) was carried out by Boglárka Mészáros and Balázs Gusztáv Mende (64). The chronology of the site is confirmed by radiocarbon analysis.

The graves were dug into a Roman pit between the southern wall of the Late Roman fort and the defensive ditch 77 m south of the wall. The two burials appeared one below the other. In the top grave, the man, oriented NW-SE and between 40 and 60 y old, was buried without any grave goods, while in the bottom grave, the man, oriented N-S and between 50 and 60 y old, was buried with rich grave goods dating to the Hun period. The burial included drink and food offerings, parts likely belonging to a riding whip (nagaika), a two-row antler comb, silver belt and shoe buckles, a double-edged sword and its accessories (buckle, sword bead), and an iron knife. The pathological lesions found on the man’s spinal column and the overdevelopment of the lower limbs and pelvis, indicating a strong musculature and muscle use, can be interpreted as a consequence of a long-term equestrian way of life. The objects placed in the warrior’s grave suggest that the deceased man likely belonged to a newly arrived group from Eastern Europe, the Hun elite, who exercised control and influence, but the provincial origins of some of the burial customs may also suggest *foederati* groups with Roman connections.

#### Hajdúböszörmény-Vidi-zug (H).

The Vidi-zug site, an archaic steppe-like saline pasture with smaller watercourses, is located in the northern part of the Great Hungarian Plain, on the border between the Hortobágy and the Hajdúhát. Two burials from the Hunnic period have been excavated here on the north-western outskirts of Hajdúböszörmény. The two graves were dug into a low kurgan built during the transitional period of the Late Copper and Early Bronze Age.

The history of the site's research dates back to the mid-1980s, when the illegal disturbance of the eastern half of the mound (kurgan) revealed finds from the Hungarian Conquest period. Following the announcement of the findings, István Fodor, archaeologist at the Hungarian National Museum, uncovered 35 graves from the Hungarian Conquest period and identified three burials lying on its side in a slightly contracted position as prehistoric. In 2023, the Hajdúsági Museum carried out an archaeological excavation in the area of the earlier excavated half of the mound in cooperation with the Déri Museum and the University of Helsinki. The primary aim of this research was to fully excavate the eastern, disturbed, partially excavated half of the mound and to find and document the central Yamnaya burial. The archaeological research yielded unexpected results. In addition to the Late Copper Age Yamnaya primary/central burial, five burials from the Scythian period, two from the Hunnic period, eight from the Avar period, and 30 burials from the Hungarian Conquest Period were unearthed. The Migration-period archaeological and anthropological materials are currently being processed by a team led by Marianna Bálint and Tamás Hajdu. The dating of the burials is supported by radiocarbon dates.

One of the graves from the Hunnic period was excavated in the central part of the kurgan. This inhumation grave was superposed and disturbed by a grave from the Hungarian Conquest period, leaving only the two legs in their original “in situ” position and the bottom of the pot placed next to the leg. A bone (antler?) carving was also recovered from the filling of the grave. The position of the legs suggests that the deceased’s body was oriented west–east.

The other burial of the Hunic period, which is the subject of current analysis, was completely intact, is located near the previous grave, to the north-east of it. The adult man buried in a wooden coffin, in a north–south oriented deep rectangular grave pit, was furnished with a belt ending in a solid gold buckle. A large iron knife and another iron tool were suspended from his belt. A gold shoe buckle was found on both his right and left ankle. Another gold buckle with no pin was found under the skull, which may have functioned as a hair ornament. At the head of the deceased, in the north-eastern corner of the grave pit, on top of the coffin, a large Murga-type wheel-thrown jar was placed.

Some special sacrificial features (pits with animal remains) have also been found buried within the kurgan, which may be related to the Hunnic-period burials.

#### Hajdúnánás-Fürj-halom-dűlő, Site 40 (H).

In the Transtisza region of the Great Hungarian Plain, approximately 25 km east of the Tisza River, Hajdúnánás Site 40 (Hajdú-Bihar County, Hungary) is situated on a long, flat sand hill, 900 m from Fürj-halom, a prehistoric kurgan. 18 graves from the late Sarmatian/Hunnic (turn of the 4th to 5th c. CE), 74 from the Gepidic (2nd half of 5th to 6th c. CE), and three from the Avar periods (7th to 8th c. CE) have been discovered here. The excavation was carried out by the Institute of Archaeological Sciences of the Eötvös Loránd University in 2004. The graves discussed here belong to the Gepidic cemetery.

The 5th- to 6th-century graves lay in two groups, on the north and south sides of the Sarmatian cemetery. There was a significant number of ACD cases among the individuals buried here. The graves were mainly poorly furnished, most often with bone combs and ceramic vessels. The relative poverty of the burials may be due to the fact that about three-quarters of the grave pits have been disturbed, most of them probably a few years after the funeral. The peripheral location of the community within the Gepidic kingdom–on the northern fringe of the settlement territory–may also have contributed to the poorer finds. The dating of the site is confirmed by radiocarbon analyses (65). For an archaeological description of the burials of the individuals reported in this study (Dataset S5).

#### Pusztataskony-Ledence, Sites 1 and 2 (H).

The Pusztataskony-Ledence site (Jász-Nagykun-Szolnok county, Hungary) stands out from the former floodplain of the eastern side of the Tisza River as a dry piece of land in a flat and wet area. Several prehistoric archaeological features, a part of a Sarmatian settlement, a Hun-period solitary grave and 17 5th- to 6th-century scattered graves were unearthed here. The excavation was carried out by the Institute of Archaeological Sciences of the Eötvös Loránd University in 2009. The anthropological analysis of the site was led by Tamás Szeniczey (66).

The burial customs and grave goods of the Hun-period grave (Site 2, Grave 270/337)—north–south orientation, crescent-shaped earring, amber bead, and gray ceramic jug—fit well into the archaeological picture of the first half of the 5th century (46). In the grave lay a 35- to 50-y-old woman with an artificially deformed skull. About 150 m from this solitary burial, other graves from the 5th to 6th centuries have been found, arranged in loose groups about 100 m long (Site 1). Among these, graves 193/237 and 212/264 were found close together. The former is a double burial of a young woman and a child. These too can be dated to the Hun period, or possibly slightly later, in the second half of the 5th century. The chronology of the site is confirmed by radiocarbon analyses. For an archaeological description of the burials of the individuals reported in this study (Dataset S5).

#### Tiszagyenda-Búszerző and Lakhatom (H).

In 2006 to 2007 a large-scale rescue excavation was led in Tiszagyenda, where the site that is roughly 1 km long, covers around 10 ha and was used in the 3th to 12th centuries, was divided in two parts for survey (the northern third as Tiszagyenda-Búszerző—2,6 ha excavated by László Kocsis and the southern part as Tiszagyenda-Lakhatom—5,5 ha by Zsuzsanna Hajnal). The extremely dense site was situated on the high bank of the Tisza directly beside the floodplain of the river, where 3,402 archaeological features were unearthed including 168 burials and 10 additional skeletons found in settlement features, and among them 102 individuals belonged to the Migration periods. The anthropological analysis was done by Zsolt Bernert and Orsolya Mateovics-László. The publication of the archaeological material is in progress: Early medieval burials from Tiszagyenda.

The Hunnic and Gepidic period is represented by 66 individuals. The dating is confirmed by radiocarbon analyses. The burials were scattered over a length of about 1 km among the houses and pits of a contemporary settlement. Among the grave goods were silver and iron fibulae with inverted foot, earrings with polyhedral pendant, mirrors, bronze, and iron belt buckles with an oval fastening plate and round ring. Later, cast bow fibulae with Kerbschnitt, a pair of bird shaped fibulae with garnets, hair pins, antler combs, and daggers (sax) came to light. There was a significant number of artificial cranial deformations in this site. For an archaeological description of the burials of the individuals reported in this study (Dataset S5).

### Archaeological Description of the Investigated Burials from the Hun-Xianbei Context at Berel in Kazakhstan.

The first excavations in the Berel necropolis date back to 2003. Since then, about 90 burials have been discovered at Berel. The first finds belong to the Pazyryk culture (4th to 2nd centuries BCE), represented by typical burial mounds (kurgans) of the Saka elite (67). Starting from 2008, burials that were not related to the elite mounds of the Sakas were found on Berel (39, 68–70). These burials belong to the later Hun-Xianbei layer (2nd to 6th centuries). Hun-Xianbei burials have diverse structures and orientations of the dead, and are characterized by the random inclusion of weapons and horses in the burials. Some of the burials were multiple. The east-west orientation of the Hun-Xianbei layer graves, the extensive use of stones in the grave structures and the burials of whole horses differ greatly from the north–south oriented solitary graves buried with horse skulls/skins in the Eurasian steppe. Certain materials of the Xiongnu-Xianbei period discovered at Berel have direct analogies with the Hun-period burial grounds of the Transbaikalia region. Most of the Berel Hun-Xianbei burials were in a simple pit, the top of which was covered by stones. In some cases, a wooden box was found inside. The inventory of male burials includes iron knives, arrowheads, leather belts, bags with bronze and silver metal inserts, and ceramic vessels. Some of the female burials contained bone combs, gold, silver, and bronze earrings, beads made from round plates encrusted by stones, waist bags, and other finds. As food for the deceased, bones of small animals were found near the human remains. However, the bones of sacrificial horses with harness encrusted by metal plates were separated from the human burials. Among the interesting finds, there were two horn plates with tamga-like signs, the meaning of which is not yet entirely clear. These specially engraved symbols were not related to tamgas or runic inscriptions that were found in Turkic monuments of the Mountain and Mongolian Altai (71). Archaeological information on the individuals analyzed in the current study is reported in Dataset S6.

### Ancient DNA Lab Work, Sequencing, and Data Processing.

For the archaeogenetic investigations petrous bones and teeth were preferentially sampled (Dataset S1). Sample preparation was performed in dedicated ancient DNA laboratory facilities of the HUN-REN RCH Institute of Archaeogenomics, Budapest, Hungary. Sample surfaces were decontaminated with UV-C light, cleaned by mechanical removal. Ca. 25 to 50 mg bone powder was gained by drilling or powdering and transferred to MPI-EVA, Leipzig, Germany. DNA extraction and subsequent laboratory steps were performed in the Ancient DNA Core Unit of the MPI-EVA. DNA was extracted from between 25 mg and 52 mg of powdered sample material using a silica-based method optimized for the recovery of short DNA fragments (72). Lysates were prepared by adding 1 mL of extraction buffer (0.45 M EDTA, pH 8.0, 0.25 mg/mL proteinase K, 0.05% Tween-20) to the sample material in 2.0-mL Eppendorf LoBind tubes and rotating the tubes at 37 °C for approximately 16 h (72, 73). Using an automated liquid handling system (Bravo NGS Workstation B, Agilent Technologies) DNA was purified from 150 µL lysate using silica-coated magnetic beads and binding buffer D as described in ref. 73. Elution volume was 30 µL. Extraction blanks without sample material were carried alongside the samples during DNA extraction.

DNA libraries were prepared from 30 µL extract using an automated version of single-stranded DNA library preparation (74) described in detail in ref. 75. *Escherichia coli* Uracil-DNA-glycosylase and *E. coli* endonuclease VIII were added during library preparation to remove uracils in the interior of molecules. Libraries were prepared from both the sample DNA extracts and the extraction blanks, and additional negative controls (library blanks) were added. Library yields and efficiency of library preparation were determined using two quantitative PCR assays (75). Libraries were tagged with pairs of sample-specific indices via PCR extension using AccuPrime *Pfx* DNA polymerase as described in ref. 75. Indexed libraries were amplified and purified using SPRI technology (76) as described in ref. 75. NEPS01-2 samples were processed entirely in Budapest, as in ref. 77. UDG-half double-stranded libraries were shotgun sequenced for 2 × 150 PE on Illumina NovaSeq and NovaSeq X Plus.

### Data Processing.

We processed the raw sequenced reads data of the newly sequenced 35 individuals via the nf-core/eager pipeline (78) (https://nf-co.re/eager). We used AdapterRemoval v2.3.1 (79) to remove adaptors and reads <30 bp. We used the Human Reference Genome Hs37d5 to perform genome mapping with bwa v0.7.17 aln/samse (80) setting “-n” and “-l” parameters to 0.01 and 1,024 respectively. We discard reads with phred mapping quality <30 with “-q” parameter in Samtools v1.9 (81). We used the MarkDuplicates function of Picard tools (https://github.com/broadinstitute/picard) to remove PCR duplicates. On a subset of 100,000 reads on the “q30” bam files, we run mapDamage v2.0 (82) to estimate the amounts of taphonomic deamination (C to T). We performed DNA contamination analyses with ANGSD v0.910 (83) for the autosomal contamination levels (applicable only for males) and with Schmutzi (84) for the mitochondrial DNA contamination in both males and females. We used Schmutzi also to reconstruct the consensus mitochondrial genome sequences and HaploGrep2 (85) to assign the mitochondrial haplogroups. Y-chromosome haplogroups were inferred calling variants only on males with Samtools v1.9’s (81) mpileup and PileupCaller (https://github.com/stschiff/sequenceTools) with the parameter “--majorityCall.” Haplogroups were assigned with yHaplo (https://github.com/23andMe/yhaplo), using the ISOGG panel v.11.349 as a reference (https://isogg.org/tree/; date of access 02 February 2023). All the individuals showed negligible levels of contamination (<5%) and typical deamination levels of ancient DNA (Dataset S1). For PCA, qpAdm and DATES analyses described in the following paragraphs, we used PileupCaller with the “--randomHaploid” parameter to call pseudohaploid genotypes on the 1240K SNP panel (https://github.com/stschiff/sequenceTools). We merged the new data with a reference genome-wide panel of 2,280 modern individuals genotyped with the microarray technology using the commercial HumanOrigins chip (86–88) and 277 previously published ancient individuals produced with the 1240K capture method or a 1240K SNPs subset of whole-genome shotgun sequence data (2, 3, 34–36, 48, 86, 87, 89–97). Published genotype data were obtained via Poseidon (https://poseidon-framework.github.io).

### Population Genomic Analyses.

On the 370 individuals’ dataset we ran Principal Component analysis using smartpca v16000 implemented in EIGENSOFT v6.0.1 package (98) setting the “lsqproject” and the “autoshrink” parameters in order to project the ancient data on the principal components calculated only with the modern populations. For modern populations we used a common set of Eurasian populations as originally in ref. 86 adapted as in ref. 35, often referred to as “the Eurasian PCA.” To formalize the East Asian or steppe admixture observed from these previous analyses, we ran f4-statistics-based ancestry modeling on 29 ancient individuals using the qpWave/qpAdm v1520 programs from the ADMIXTOOLS package (88). We calculated f4-statistics on the 1240K dataset and used the default option “allsnps: NO” to use SNP overlap between all the groups for each test. SE were estimated through block jack-knifing using a 5 cM block. We used a set of Eurasian source groups (or left populations) from reference genomic data from the Bronze Age to the Early Middle Ages (Dataset S2) and the following outgroup set (or right populations) which are representatives of key Eurasian lineages: central African rainforest hunter-gatherers (Mbuti, n = 4), Andamanese islanders (Onge, n = 2), European Mesolithic hunter-gatherers (Iron_Gates_HG, n = 40) (93), eastern European hunter-gatherers (EHG, n = 4) (92, 99), Neolithic Iranians (Iran_N, n = 7) (87, 94), Neolithic Anatolians (Anatolia_N, n = 30) (92), Neolithic Levantines (Levant_N, n = 6) (87), Neolithic Longshan individuals from Yellow River basin (YR_LN, n = 8) (48), Bronze Age agro-pastoralists from the Tarim Basin (Tarim_EMBA1, n = 12) (95), and Bronze Age pastoralists from the northern Mongolia (Mongolia_Khovsgol_LBA, n = 19) (90). The results of all the tests performed are reported in Dataset S3. We then performed admixture dating with DATES v.753 (https://github.com/priyamoorjani/DATES) to date the average time of the East-West Eurasian ancestry admixture following the same procedure as in ref. 42. We used the un-admixed and high SNP covered LBA/IA group of Ulaanzuukh_SlabGrave in Mongolia as ANA proxy and the pre-Avar Carpathian Basin ancient sources, then Sarmatian and Longobard period individuals as West Eurasian ancestry proxy (Local_Sarmatian_Langobard). In the case of HDB001 given the qpAdm modeling results we also tested an admixture date between an Iranian related source: Bustan_BA (Dataset S4). The time ranges and their average point used to anchor the time for the admixture dating of each individual were chosen based on the evaluation of the archaeological chronology.

To perform pairwise haplotype IBD analysis, we first called genotype likelihoods (GLs) on the “q30” bam files with ATLAS (https://bitbucket.org/wegmannlab/atlas/) (100) using the “MLE” function after performing base quality recalibration with the “recal” function and assessing the postmortem deamination pattern with the “PMD” function. The recalibration step also corrects for the empirical ancient deamination pattern and reduces the effect of reference bias by correcting with a list of 10 million highly conserved genomic positions across 88 mammal species (https://grch37.ensembl.org/). We called the ~20 M SNP positions covered on the phased 1000 Genomes Phase 3 release data (1KG) as reference haplotypes (101) that were also used as the reference panel for the imputations. We run GLIMPSE (102) for genome-wide imputation using default parameters and sex-averaged genetic maps from HapMap on genomic chunks of 2,000,000 bp with a buffer of 200,000 bp. After obtaining the phased genotypes with their posterior probabilities from GLIMPSE, we performed Haplotype-IBD analysis with ancIBD (52). We included only individuals with more than 450 k SNPs obtained with our pseudohaploid calls and SNPs with GP > 0.99 after imputation. We used the “HapBLOCK” function of ancIBD with default parameters, including only shared blocks of more than 8 cM containing >220 SNPs per centimorgan. As recommended by the developers we considered only IBD segments ≥8 cM and performed additional filtering for specific figures as specified in the figures legends. We used Cytoscape v3.9.1 (103) to plot the networks of pairwise IBD relations.

### IBD Modeling Analyses.

We found many IBD sharing between 5th and 6th centuries Carpathian Basin individuals and late Xiongnu period individuals, including long IBD segments (>12 cM) that indicate recent genealogical connections (*SI Appendix*, Fig. S2). To quantify the time depth of this connection, we used the two-island split model (*SI Appendix*, *Supporting Information Text*). It assumes that the two populations of interest (here the Hun group and late Xiongnu group) stem from a common ancestral population and there is no subsequent gene flow after the split (*SI Appendix*, Fig. S3*A*). We used 36 late Xiongnu individuals with a median date of 25CE (50BCE to 100CE) (referred below as the late Xiongnu group) and 17 Hun period individuals exhibiting various levels of East Asia ancestry with a median date of 519CE (470 to 567CE) (referred below as the Hun group). Assuming a generation time of 29 y, the Huns and late Xiongnu are temporally separated by ~17 generations.

We fitted the model by maximum likelihood (*SI Appendix*, *Supporting Information Text*) and computed the CI by doing a grid search over a dense 2D grid to find combinations of N_0_ and T_0_ whose log likelihood is no smaller than the maximum likelihood minus 1.92 (*SI Appendix*, Fig. S3*D*). We estimated the split time to be 18 generations (95% CI: 17 to 21, the time is measured with respect to the date of the Hun group) and the ancestral population size to be 34,245 (95% CI: 20,750 to 40,600). Because the estimated split time is almost the same as the date difference between the Hun and late Xiongnu groups, this suggests that either late Xiongnu group is the direct source of the Hun group or it is genealogically connected to the source of the Hun group by at most a few hundred years. The fitted model predicts the empirical IBD reasonably well (*SI Appendix*, Fig. S3*B*). However, we observe that the model consistently underestimates the long IBDs (*SI Appendix*, Fig. S3*C*). This indicates that there might be recent, continuous gene flow between the late Xiongnu and the Hun groups that cannot be accommodated by the simple model fitted here and this would lead to an underestimate of the split time. Nevertheless, taken altogether, the presence of long IBD sharing between the Hun and late Xiongnu groups and the genetic split time estimates, suggest that the ancestors of the Hun group are at most a few hundred years away from the late Xiongnu group.

## Supplementary Material

Appendix 01 (PDF)

Dataset S01 (XLSX)

Dataset S02 (XLSX)

Dataset S03 (XLSX)

Dataset S04 (XLSX)

Dataset S05 (XLSX)

Dataset S06 (XLSX)

## Data Availability

The produced sequence data are deposited in the European Nucleotide Archive (ENA) with the following accession number: PRJEB79921 (104). The haploid genotype data are available through the Poseidon framework under: 2025_GnecchiRuscone_CarpathianBasinHunPeriod (105).
