# Supplementary material for: Ancient genomes reveal trans-Eurasian connections between the European Huns and the Xiongnu Empire
Source: Proc Natl Acad Sci U S A. 2025 Feb 24;122(9):e2418485122. doi: 10.1073/pnas.2418485122 (PMC11892651; doi:10.1073/pnas.2418485122)
Supplement: Supplementary file 1 — Appendix 01 (PDF) [file pnas.2418485122.sapp.pdf]

Ancient Genomes reveal trans-Eurasian connection between European Huns and the Xiongnu Empire

Guido Alberto Gnecchi-Ruscone*,#,1,2, Zsófia Rácz*,#,3, Salvatore Liccardo[4,5], Juhyeon Lee[6,7], Yilei Huang[1], Luca Traverso[1], Rita Radzeviciute[1], Zsuzsanna Hajnal[8], Anna Szécsényi-Nagy[9,10], Balázs Gyuris[9,11], Orsolya Mateovics-László[12], Zsolt Bernert[13], Tamás Szeniczey[14], Tamás Hajdu[14], Boglárka Mészáros[15], Marianna Bálint[16], Balázs Gusztáv Mende[9], Bryan Miller[17,18], Samashev Zainolla[19,20], Ainash Childebayeva[1,21], Leyla Djansugurova[22], Patrick Geary[23], Harald Ringbauer[1], Tivadar Vida[3,24], Choongwon Jeong[6,7], Walter Pohl#,4,5, Johannes Krause#,1 & Zuzana Hofmanová#1,2

[1]Department of Archaeogenetics, Max Planck Institute for Evolutionary Anthropology, 04103, Leipzig, Germany
[2]Department of Archaeology and Museology, Faculty of Arts, Masaryk University, 60200, Brno, Czechia
[3]Institute of Archaeological Sciences, ELTE - Eötvös Loránd University, 1088 Budapest, Hungary
[4]Institute of Austrian Historical Research, University of Vienna, 1010 Wien, Austria
[5]Institute for Medieval Research, Austrian Academy of Sciences, 1010 Wien, Austria
[6]School of Biological Sciences, Seoul National University, Seoul 08826, Republic of Korea
[7]Institute for Data Innovation in Science, Seoul National University, Seoul 08826, Republic of Korea
[8]Hungarian National Museum,1088 Budapest, Hungary
[9]Institute of Archaeogenomics, HUN-REN Research Centre for the Humanities, 1097 Budapest, Hungary
[10]MTA-BTK Lendület "Momentum" Bioarchaeology Research Group, 1097, Budapest, Hungary
[11]Doctoral School of Biology, ELTE - Eötvös Loránd University, 1117 Budapest, Hungary
[12]Archäologischer Dienst GesmbH, 3100 St. Pölten, Austria
[13]Department of Anthropology, Hungarian Natural History Museum, Ludovika tér 2-6, 1083 Budapest, Hungary
[14]Department of Biological Anthropology, ELTE - Eötvös Loránd University, 1117 Budapest, Hungary
[15]Budapest History Museum, Aquincum and Archaeological Park, 1031 Budapest, Hungary
[16]Hajdúsági Museum, 4220 Hajdúböszörmény, Hungary
[17]Museum of Anthropological Archaeology, University of Michigan, Ann Arbor, MI 48109, USA
[18]History of Art, University of Michigan, Ann Arbor, MI 48109, USA
[19]State Historical and Cultural Museum-Reserve "Berel", Zhambyl, 070906, Kazakhstan.
[20]Branch of Institute of Archaeology by A.Kh. Margulan, 010011 Nur-Sultan, Kazakhstan
[21]Department of Anthropology University of Texas at Austin, Austin, TX 78712, USA
[22]Center of Paleogenetics and Ethnogenomics, Institute of Genetics and Physiology, 050060, Almaty, Kazakhstan
[23]Institute for Advanced Study, Princeton, NJ 08540, USA
[24]Institute of Archaeology, HUN-REN Research Centre for the Humanities, 1097 Budapest, Hungary

*These authors contributed equally to this work
#Corresponding authors: guido_gnecchi@eva.mpg.de; zsofia_racz@yahoo.de; walter.pohl@oeaw.ac.at; krause@eva.mpg.de; zuzana_hofmanova@eva.mpg.de

This PDF includes:

Supporting Information Text

Figures S1 to S3

Other supporting materials for this manuscript include the following

Datasets S1 to S6

Supporting Information Text

S1. Modelling of the split-time between Late Xiongnu and Hun

Yilei Huang & Harald Ringbauer

1. Method

1.1 Model Specification

To estimate genetic split times from shared IBD segments, we use the framework presented in (1). Given a demographic model, it computes the expected rate of shared IBD segments of length l by integrating over time the product of single-locus coalescent rate (denoted by $\phi(t)$) and the average number of genomic blocks of length l (denoted by $E[K_l^t]$). As in (1), we assume a simple two-island split model with no subsequent gene flow (see Supplementary Figure 3a). For this model, the single-locus coalescent rate is:

$$\phi(t) = 0, t < T_0, \frac{1}{2N_0} e^{-\frac{t-T_0}{2N_0}}, t \geq T_0 \quad (1)$$

where T_0 denotes the split time and N_0 is the (diploid) effective population size of the common ancestral population.

The model described in (1) assumes that the genetic data from both islands are collected at the same generation. However, this is not necessarily true for ancient DNA. To account for this time difference, here we modify $E[K_l^t]$ to model the situation where the genomes from two islands differ by a time difference of δt generations (we require that $\delta t < T_0$):

$$E[K_l^t; \delta t] = 2 \cdot (2t - \delta t) \cdot exp(-l(2t - \delta t)) + (G - l) \cdot (2t - \delta t)^2 \cdot exp(-l(2t - \delta t)). (2)$$

where G is the map length of a chromosome.

Integrating the product of $\phi(t)$ and $E[K_l^t; \delta t]$over t gives:

$$E[K_l] = -\frac{e^{l(\delta_t - 2T_0)}}{(4lN_0 + 1)^3}(-(G - l)(\delta_t + 4\delta_t lN_0)^2 + 2\delta_t(4lN_0 + 1)(2T_0(G - l)(4lN_0 + 1) + 4GN_0 + 1)(G - l)(4lN_0T_0 + T_0)^2 - 4T_0(4GN_0 + 1)(4lN_0 + 1) - 8N_0(4GN_0 + 1))$$

(3)

We note that for $\delta t = 0$, Eq.3 reduces to Eq.~4 in (1). The remainder of the inference of split times is as described in (1).

1.2 Modelling IBD Detection Errors

Detecting IBD segments is not perfect. To account for errors of IBD segments in empirical aDNA data, we use the same error model for IBD detection as applied in (2,3,4). Briefly, the error model considers three sources of IBD detection errors: false positive, power, and length bias. Let $\lambda(y)$ and $\hat{\lambda}(y)$ denote the true and empirically inferred rate of IBD sharing of length y, respectively. Then:

$$\hat{\lambda}(y) = FP(y) + \int_0^\infty \lambda(z) Power(z) R(y|z) dz$$

where $FP(y)$ is the false positive rate of IBD block of length y, $Power(y)$ is the power to detect a true segment of length z and $R(y|z)$ is the probability that a segment of true length z is detected being length y.

We numerically estimated the parameters of the error model from simulations mimicking typical aDNA data described in Supplementary Note 2 in (4).

References

1. Yilei Huang, Shai Carmi, David E Reich, and Harald Ringbauer. Genetic estimates of the initial peopling of polynesian islands actually reflect later inter-island contacts. *bioRxiv*, 2022–12, 2022.
2. Peter Ralph and Graham Coop. The geography of recent genetic ancestry across europe. *PLoS biology*, 11(5):e1001555, 2013.
3. Harald Ringbauer, Graham Coop, and Nicholas H Barton. Inferring recent demography from isolation by distance of long shared sequence blocks. *Genetics*, 205(3):1335–1351, 2017.
4. Huang, Yilei, Shai Carmi, and Harald Ringbauer. "Estimating effective population size trajectories from time-series Identity-by-Descent (IBD) segments." *bioRxiv* (2024): 2024-05.

Supplementary figures

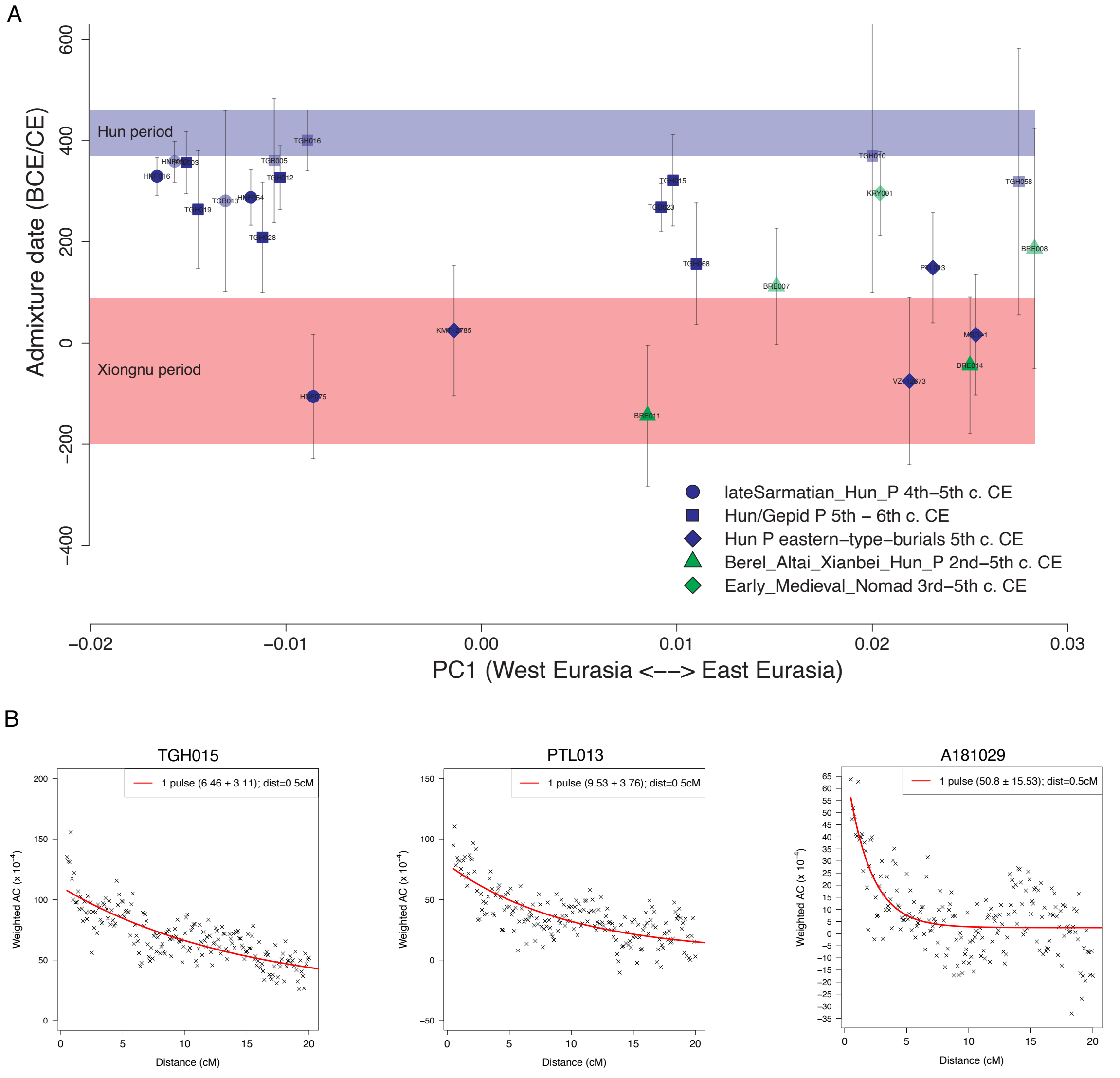

Fig. S1. Admixture dating results. A. On the x axis are plotted the PC1 coordinates (of PCA reported in Fig. 2A). On the y axis the admixture dates obtained with DATES are converted into years BCE (negative values) and CE (positive values) between West and East Eurasian sources. A transparency factor is added to the date estimates with z-scores < 2. B. Weighted ancestry covariates decay plots for three individuals as an example of two recent admixture dates (TGH015, with standard error fall within the Hun period and PTL013, pre-dating the Hun period) and A181029 that shows a much older admixture date, predating Xiongnu period and outside of the lower range of panel A.

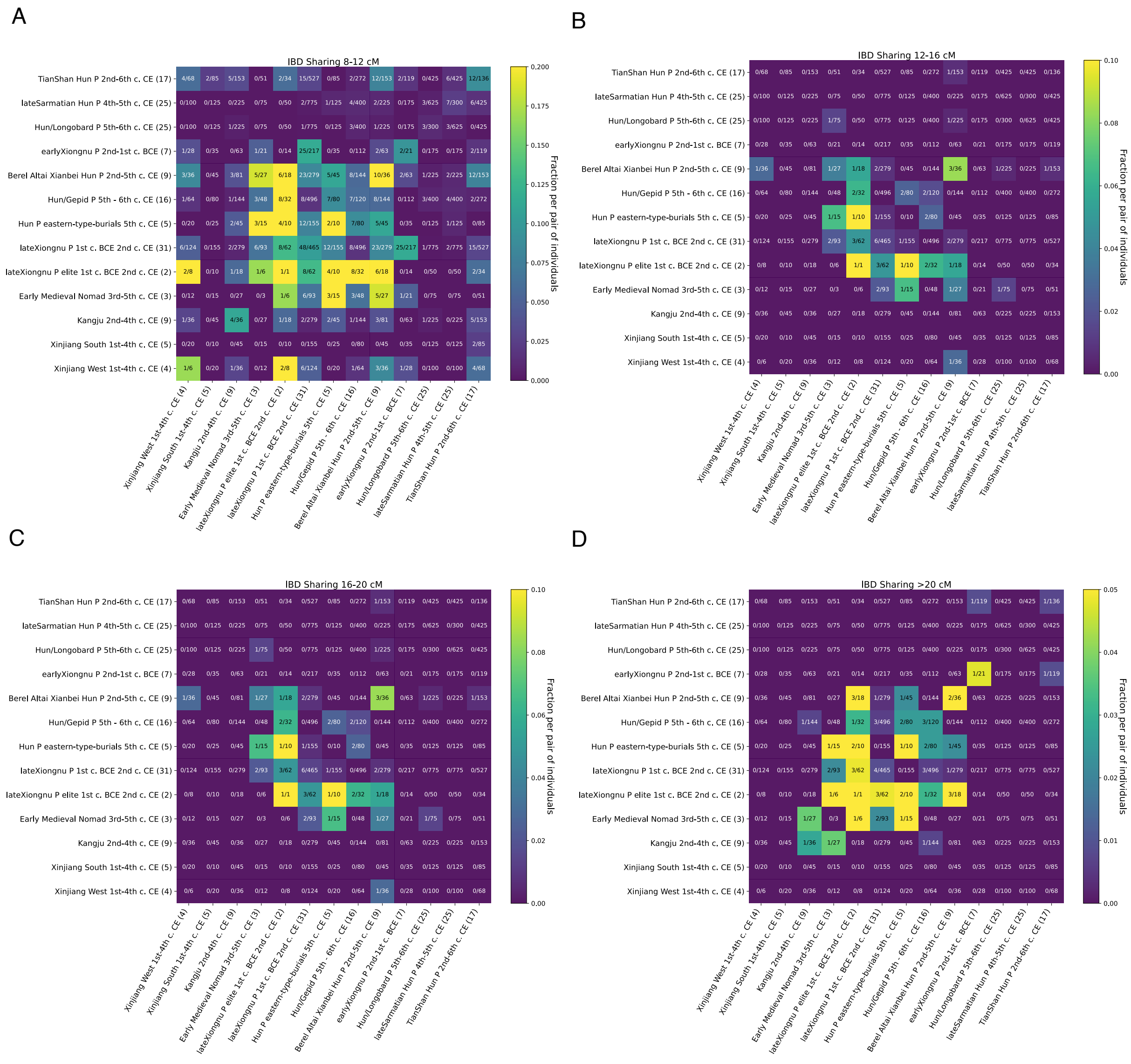

Fig. S2. Matrices of IBD sharing between the archaeological groups analyzed in the study. A-D four length bins are shown in each panel: 8-12 cM, 12-16 cM, 16-20cM and >20cM. The color gradient reflects the fraction of number of IBD shared over the total possible number of pairs given the number of individuals inside each group (reported in brackets next to the label of the group). We used the same archaeological groups as in the legends of Fig. 2A.

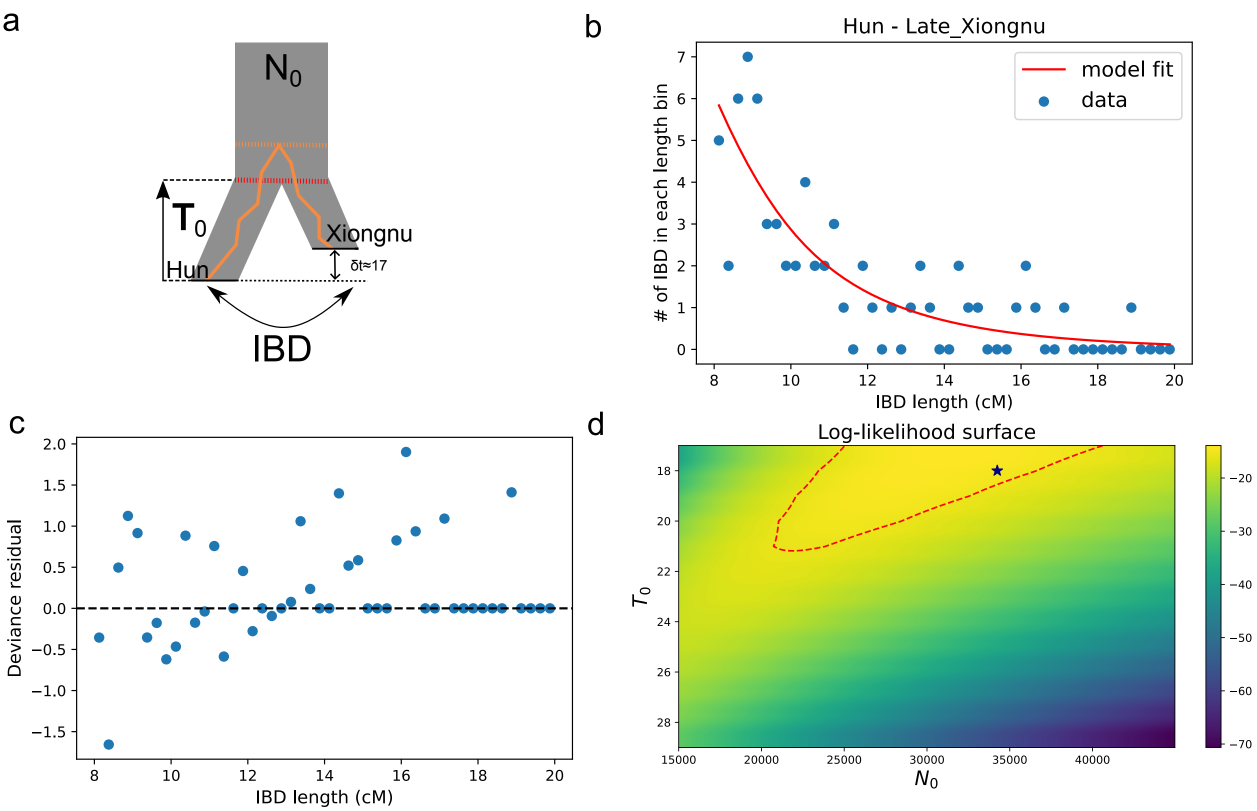

Fig. S3. IBD modeling between late Xiongnu period and the Hun/Gepid period individuals a. A sketch of the two-island split model. It assumes that two populations (here Hun and Xiongnu) stem from the same ancestral population (with effective population size N_0) T_0 generations ago. Samples from the two populations might be taken at different times, separated by δt generations. IBD segments shared between samples from the two populations are used to infer T_0 and N_0. b. Observed IBD segments (plotted as blue dots in bins of width 0.25cM) versus predicted IBD sharing from the fitted two-island split model. c. Deviance residual of the fitted model in each length bin of width 0.25cM. d. Log-likelihood of the model across a dense 2D grid of T_0, N_0.

Legend Datasets

Datasets S1. Sequencing information of the newly sequenced data produced for this study.
Datasets S2. Reference dataset. All published and new individuals analysed in the study.
Datasets S3. Ancestry deconvolution qpWave/qpAdm analyses.
Datasets S4. Admixture dating DATES analyses.
Datasets S5. Archaeological characteristics of individuals with East Asian admixture from 5th-6th century cemeteries newly sequenced for this study.
Datasets S6. Archaeological characteristics of individuals from the Hun-Xianbei context at Berel site analyzed in this study.
